# On the Proper Age for Females to Marry

**Published:** 1844-07-27

**Authors:** 


### ON THE PROPER AGE FOR FEMALES TO MARRY.

*M. Raciborski*, in his recent elaborate memoirs on this subject, makes the following curious observations:

"*M. Marc* says—and we think that he is quite right in the statement—that the strength and the vigor of the offspring are more dependent upon the state of the mother's than of the father's constitution. The eggs, for example, of very young hens are always small, however lusty be the cock that has fecundated them. The same holds good in the case of calves, colts, &c.

According to the tables in the late Mr. Sadler's work, the average offspring of each marriage in England, when the mother is below 16 years of age, is 4,40; when her age is from 16 to 20, it is 4,63; when from 20 to 23, it is 5,21; and when from 24 to 27, it is 5,43. If these calculations be correct, they afford the most convincing evidence, that, not only the number, but also the strength and *viability*, of children born, are much influenced by the age of the mother. According to our views, the interval between the 20th and the 24th years is the most advisable for marriages among the women of this country (France.) The testimony of an accomplished female writer on the education of young persons may be aptly quoted here. "We are in the habit of marrying our daughters so young," says *Madame de Remusat*, "that they really have not had the time to look at or understand anything in its proper light. If established usages could be suddenly broken through, and if we were to consult nature more in our matrimonial arrangements, I believe that the age of 25 years, or so, is that which would be considered most advisable for young women to marry. But, alas! there is little hope that fashion will recognise so great a change in her customs as this. We should at least wait till a girl has passed her 20th year; and meanwhile everything should be done, by a judicious system of education, to hasten on the maturity of her reason."—*Ibid.*

